# ESTs Analysis Reveals Putative Genes Involved in Symbiotic Seed Germination in *Dendrobium* *officinale*

**DOI:** 10.1371/journal.pone.0072705

**Published:** 2013-08-13

**Authors:** Ming-Ming Zhao, Gang Zhang, Da-Wei Zhang, Yu-Yun Hsiao, Shun-Xing Guo

**Affiliations:** 1 Institute of Medicinal Plant Development, Chinese Academy of Medical Sciences & Peking Union Medical College, Beijing, People’s Republic of China; 2 Medical Laboratory College, Beihua University, Jilin, People’s Republic of China; 3 Department of Life Sciences, Orchid Research Center, National Cheng Kung University, Tainan, Taiwan; University of Kent, United Kingdom

## Abstract

*Dendrobium* *officinale* (Orchidaceae) is one of the world’s most endangered plants with great medicinal value. In nature, *D* *. officinale* seeds must establish symbiotic relationships with fungi to germinate. However, the molecular events involved in the interaction between fungus and plant during this process are poorly understood. To isolate the genes involved in symbiotic germination, a suppression subtractive hybridization (SSH) cDNA library of symbiotically germinated *D* *. officinale* seeds was constructed. From this library, 1437 expressed sequence tags (ESTs) were clustered to 1074 Unigenes (including 902 singletons and 172 contigs), which were searched against the NCBI non-redundant (NR) protein database (E-value cutoff, e^-5^). Based on sequence similarity with known proteins, 579 differentially expressed genes in *D* *. officinale* were identified and classified into different functional categories by Gene Ontology (GO), Clusters of orthologous Groups of proteins (COGs) and Kyoto Encyclopedia of Genes and Genomes (KEGG) pathways. The expression levels of 15 selected genes emblematic of symbiotic germination were confirmed via real-time quantitative PCR. These genes were classified into various categories, including defense and stress response, metabolism, transcriptional regulation, transport process and signal transduction pathways. All transcripts were upregulated in the symbiotically germinated seeds (SGS). The functions of these genes in symbiotic germination were predicted. Furthermore, two fungus-induced calcium-dependent protein kinases (CDPKs), which were upregulated 6.76- and 26.69-fold in SGS compared with un-germinated seeds (UGS), were cloned from *D* *. officinale* and characterized for the first time. This study provides the first global overview of genes putatively involved in *D* *. officinale* symbiotic seed germination and provides a foundation for further functional research regarding symbiotic relationships in orchids.

## Introduction

Orchidaceae, which comprises an estimated 20000 to 35000 species, constitutes one of the most diverse families [1], and most species of this family have medicinal and ornamental properties [2]. Orchidaceae are among the most evolutionarily and ecologically significant plants and are known for a wide variety of epiphytic and terrestrial growth forms and hardiness; they successfully colonize almost every habitat on earth, including soil (terrestrial), rock surfaces (lithophytic) and other plants (epiphytic). Almost all orchids seeds are extremely small (0.3-14µg), dust-like and contain notably few nutrient reserves [3]. Lacking nutrients for early seedling development, orchids are dependent on a supply of carbohydrate and other nutrients derived from mycorrhizal fungal hyphae during the achlorophyllous seedling stage [4]. In parallel, fungal hyphae invade and colonize the orchid root cortex and subsequently form elaborate intracellular coils called pelotons, which are structures distinctive in both appearance and distribution patterns with orchid tissues [5]. Several species of fungi that engage in symbiosis and provide nutrients, such as nitrogen (N) and carbon (C), during orchid seed germination have been isolated and characterized. Most of these species have been classified as orchid-associated Rhizoctonia-type fungi [6,7], in which Tulasnellaceae, Sebacinaceae and Ceratobasidiaceae have been reported to support seed germination and protocorm development [6,8,9].

In recent years, research has been conducted on the determinants of symbiosis between orchids and mycorrhizal fungi. Studies on morphological features have revealed that along with the mycorrhizal fungi that colonize tissues, the nuclei of infected cells of a mycorrhizal protocorm are visibly hypertrophied [10]. These cells display elevated DNA content [11,12], and the starch grains within the cell have vanished [13]. In parallel, with the formation of complex pelotons, a series of biochemical reactions, including signaling, ROS, and Ca^2+^… etc. are systemically induced [14], and polysaccharides, inorganic salts, trace elements and secondary metabolites subsequently accumulate [15]. However, the molecular events associated with these processes remain poorly understood. Studies on other types of mycorrhizal symbiosis at the molecular level have made some progress. In arbuscular mycorrhizal (AM) symbiosis, functions of many genes that are essential for the various stages of nodule development have been elucidated in two model legume species, *Medicago truncatula* and *Lotus* *japonicas* [16]. AM symbiosis has been shown to be associated with root nodule symbioses (RNS), and a common pathway in AM endosymbiosis in legumes has been established [17]. Seven known AM-associated genes (*CASTOR, SymRK, POLLUX, NUP133, NUP85, Cyclops* and *CCaMK*) were shown to be recruited during the evolution of RNS [18]. Comparatively, the molecular mechanisms of orchidaceae symbiotic seed germination are indistinct. The only report regarding orchid mycorrhizal (OM) symbiosis describes the characterization of gene expression in roots of *Cypripedium* *parviflorum* incubated with a mycorrhizal fungus. However, only two genes encoding trehalose-6-phosphate synthase phosphatase (*Tps*) and nucleotide binding protein (*NuBP*) were identified and cloned [19]. Therefore, there is an urgent need for a clearer picture of gene expression in OM symbiosis.

*D* *. officinale* , a representative species of orchidaceae with great medicinal value, was selected for further analysis in this study. *D* *. officinale* , which has been officially recorded in Chinese pharmacopoeia, contains a number of effective components, including sesquiterpene fluorine ketone, dibenzyl phenols, philippines phenols, alkaloids and polysaccharides. *D* *. officinale* has been used in traditional Chinese medicine as a Yin tonic to nourish the stomach, relieve throat inflammation and fatigue, promote the secretion of body fluids, reduce peripheral vascular obstruction, prevent the development of cataracts and enhance the immune system. However, limited natural resources and high demand threaten the survival of the species, which has been listed as an endangered species and catalogued in the Chinese Plant Red Book since 1987 [8]. The low germination rate under natural conditions is the main factor that limits *D* *. officinale* propagation and its protection [20]. Revealing the mechanisms of germination would be beneficial for both protecting germ plasm resources and improving germination rates in nature. The objective of this work was to assess the genetic basis of symbiotic germination in orchid seeds. To understand the mechanisms of this process better at the molecular level, an SSH cDNA library of symbiotically germinated *D* *. officinale* seeds was constructed. Differentially expressed genes were examined by sequencing and analyzing ESTs present in the library. Because symbiosis is the basis of seed germination, the functions of these genes were predicted, and the genes were classified as symbiosis-related genes or germination-associated genes. The collection of identified genes presented in the study provides an important genomic resource for future molecular studies on symbiotic seed germination in *D* *. officinale* and other OM symbiosis.

## Materials and Methods

### Plant material preparation

The capsules of *D* *. officinale* were collected from Meng-hai experimental base of Yunnan branch, Institute of Medicinal Plant Development, Chinese Academy of Medical Sciences & Peking Union Medical College Xishuanbanna, Yunnan, China. Our plant materials gathered from experimental base, and did not require specific permission. The corresponding author will be responsible for it. *D* *. officinale* is an endangered species; our purpose of this experimente is to reveal the mechanism of seed germination in nature conditions and to better protect the endangered species.

Nine capsules from eight maternal *D* *. officinale* growing on *Quercus* were collected (November, 2011) and divided into two groups. One group of seeds was cultured with *Sebacina* sp., which was isolated from *D* *. officinale* symbiotic seeds [8] (Genbank number EU910926 and public culture collection accession number CGMCC No. 3398) on oatmeal agar (OMA) medium [21] was germinated to the third stage, which is characterized by the appearance of protomeristem [22]. These seeds were defined as SGS. The other group defined as UGS was obtained by culturing *D* *. officinale* seeds on OMA medium without fungi. Seeds were sown according to the procedures described by Stewart et al. (2003) [23] and incubated in a high-humidity (75±5%) chamber at 25±2°C for a 16 h light (1500-2000 Lx) period and 8 h dark period for 5 weeks. Tissue-cultured seedlings (stored at the National Engineering Laboratory for Breeding of Endangered Medicinal Materials, Institute of Medicinal Plant Development, Chinese Academy of Medical Sciences & Peking Union Medical College) were carefully transferred to the 20 cm diameter pots and cultured in growth media (bark: pebble: coarse humus=3:1:1) in a high-humidity (75±5%) chamber at 25±2°C for a 16 h photoperiod with a light density of 400-1000 Lx for two months for aseptic tissue collection. All plant materials, including intact root, leaf, stem, UGS and SGS were collected, immediately frozen in liquid nitrogen and stored at -80° C prior to RNA extraction. A fraction of SGS and UGS were fixed in 2.5% glutaraldehyde (final concentration: 0.1M) for morphological observation.

### Total RNA extraction and SSH library construction

100 mg tissues of each sample including SGS, UGS, root, stem and leaf were gathered to extract total RNA using the EASYspin Plus Kit (Aidlab, Beijing, China) according to the manufacturer’s recommendations. Genomic DNA was removed with RQ_1_ RNase-free DNase (Promega, Madison, WI, USA). The quality and quantity of total RNA were assessed using a 1.2% formamide denaturing gel using an RNA ladder (Invitrogen, Carlsbad, CA, USA) and a NanoDrop^TM^ 2000 spectrophotometer (Thermo, Fisher Scientific, Waltham, MA, USA), respectively. cDNA was synthesized via reverse transcription using M-MLV Reverse Transcriptase kit (Promega, Madison, WI, USA) and stored at -20° C. A “forward” subtraction library was constructed using the PCR-Select ^TM^ cDNA Subtraction Kit (Clontech, Palo Alto, CA, USA). The tester and driver cDNA populations were generated from total RNA extracted from SGS and UGS, respectively. The final PCR products were purified using a QIAquick PCR purification Kit (Qiagen, Hiden, Germany) and directly cloned into the pGEM-T Easy vector (Promega, Madison, WI, USA), which was then transformed into competent *Escherichia coli* DH5α cells (Trans, Beijing, China). Transformed cells were grown on standard LB/ampicillin/X-gal/IPTG plates at 37°C for blue/white colony screening.

### EST assembly, annotation and functional classification

Positive recombinant plasmids were sequenced by Beijing Genewiz, Inc. The resulting sequences were trimmed to eliminate vectors and adaptor sequences with Codoncode Aligner 4.0.4 (CondonCode Corporation, Dedham, MA) and were subsequently assembled into unique sequences (containing contigs and singletons) with the CAP3 Sequence Assembly Program [24]. The insert sequences were manually assessed for similarities against the NCBI non-redundant protein database (NR) using BLASTX after translating DNA sequence into the respective amino acid sequence in six frames. ESTs with significant database matches (E-value ≤ 1e-5) were classified into functional categories according to the Gene Ontology database (http://www.geneontology.org), and gene expression was analyzed via EST sampling using COG database (http://www.ncbi.nlm.nih.gov/COG/). Pathway assignments were mapped according to the Kyoto Encyclopedia of Genes and Genomes (KEGG) database (http://www.genome.ad.jp/kegg/kegg2.html) (version KEGG 50) [25].

### Real-time qPCR analysis

To verify the quality of the SSH library and analyze the characterization of gene expression in fungi-infected plant tissues, 15 Unigenes were selected from different functional classes and their expression level were determined in SGS and UGS. Real-time qPCR technique were also used to determine the tissue expression pattern of two fungus-induced *CDPK* genes in non-infected plant tissues including UGS, root, stem and leaf, and their possible physiological functions were estimated. Using 2 µg of total RNA isolated from each sample as a template, reverse transcription reaction was performed (20 µL total volume) according to the Moloney Leukemia Virus Reverse Transcriptase (M-MLV RT) (Promega, Madison, WI, USA) kit protocol. The cDNAs were diluted 1:40 in ddH_2_O before performing the qPCR assay. Amplification curves were generated for all gene-specific primers designed using primer 5 (Table S1). Real-time qPCR was performed using a real-time SYBR green kit (Takara, Dalian, China) and the ABI 7500 real-time PCR System (Applied Biosystems, Foster City, CA, USA). The assay mix (final volume 25 μL) contained 12.5 µL 2× SYBR^®^ Premix *Ex Taq* ^TM^ Master Mix (Takara Bio, Dalian, China), 0.5 µL each primer (10 µM), 0.5 µL ROX Reference Dye, 2 µL cDNA template, and 9 µL ddH_2_O. The thermal cycling conditions were as follows: 95° C for 30 s, followed by 40 cycles of 95° C 15 s and 60° C 40 s. The relative expression was calculated as the ratio of target gene expression normalized to *GAPDH* expression [26]. All standards were amplified in duplicate, and samples were amplified in triplicate. All qPCR products were subjected to melting curve analysis and verified via gel electrophoresis. Cycle threshold (Ct) values were generated using the ABI PRISM 7500 Software Tool (Applied Biosystems, Foster City, CA, USA), and the relative expression ratios were calculated using the comparative 2^-∆∆CT^ method of relative gene quantification [27].

### Cloning and characterization of two full-length CDPK genes

Isolation of the 3'/5’-end of the two *CDPK* genes was performed using a SMART™ RACE cDNA Amplification Kit (Clontech, Japan) according to the manufacturer’s instructions. Based on the sequences of the SSH cDNA Library, the gene-specific primers (GSP) DoCDPK1- R/F and DoCDPK32-like- R/F (Table S1) were designed. A thermal profile was obtained on a ABI-Veriti™ 96-well thermo cycler (Applied Biosystems, Foster City, CA, USA), and the assay mix (25 µL reaction volumes) contained 2.5 µL 10× Advantage^®^ 2 PCR buffer, 0.5 µL dNTPs (10 mmol·L^-1^), 0.5 µL DoCDPK1-R/F or DoCDPK32-like-R/F（10 µmol•L^-1^）, 0.5 µL UPM（10 µmol·L^-1^), 1.0 µL 3'/5’-RACE ready cDNA, 0.5 µL 50× Advatange^®^ 2 Polymerase Mix（5 U·µL^-1^) and 19.5 µL ddH_2_O. The PCR amplification program consisted of a 2 min activation step followed by 40 cycles of denaturation for 20 s at 95° C, annealing for 30 s at 68° C, and extension for 1.5 min step at 72° C and was terminated with an extension step for 7 min at 72° C. The amplified products were purified, cloned into a pGEM-T Easy vector (Promega, Madison, WI, USA) and transformed into competent DH5α cells. Positive recombinant plasmids were screened with AMP/IPTG/X-gal media and sequenced by Beijing Genewiz, Inc. After clustering and splicing with the core sequence, the ORFs of each sequence were obtained using BLASTX and ORF finder. Primers (Table S1) targeting the ORF determined in RACE screens were used to sequence and confirm the first reconstituted RACE sequences. The nucleotide sequences of *DoCDPK1* and *DoCDPK32-like* reported in the current study are available in the GenBank database under the accession numbers JX002662 and JQ927225, respectively. Sequences were aligned using MegAlign in the Lasergene software package of DNAstar 6.0 (Madison, WI, USA). A neighbor-joining phylogenetic tree was generated using MEGA 4.0 software package (version 4.0, http://www.megasoftware.net, Tempe, AZ, USA) with default parameters.

## Results

### Morphological examination

**Figure 1:**
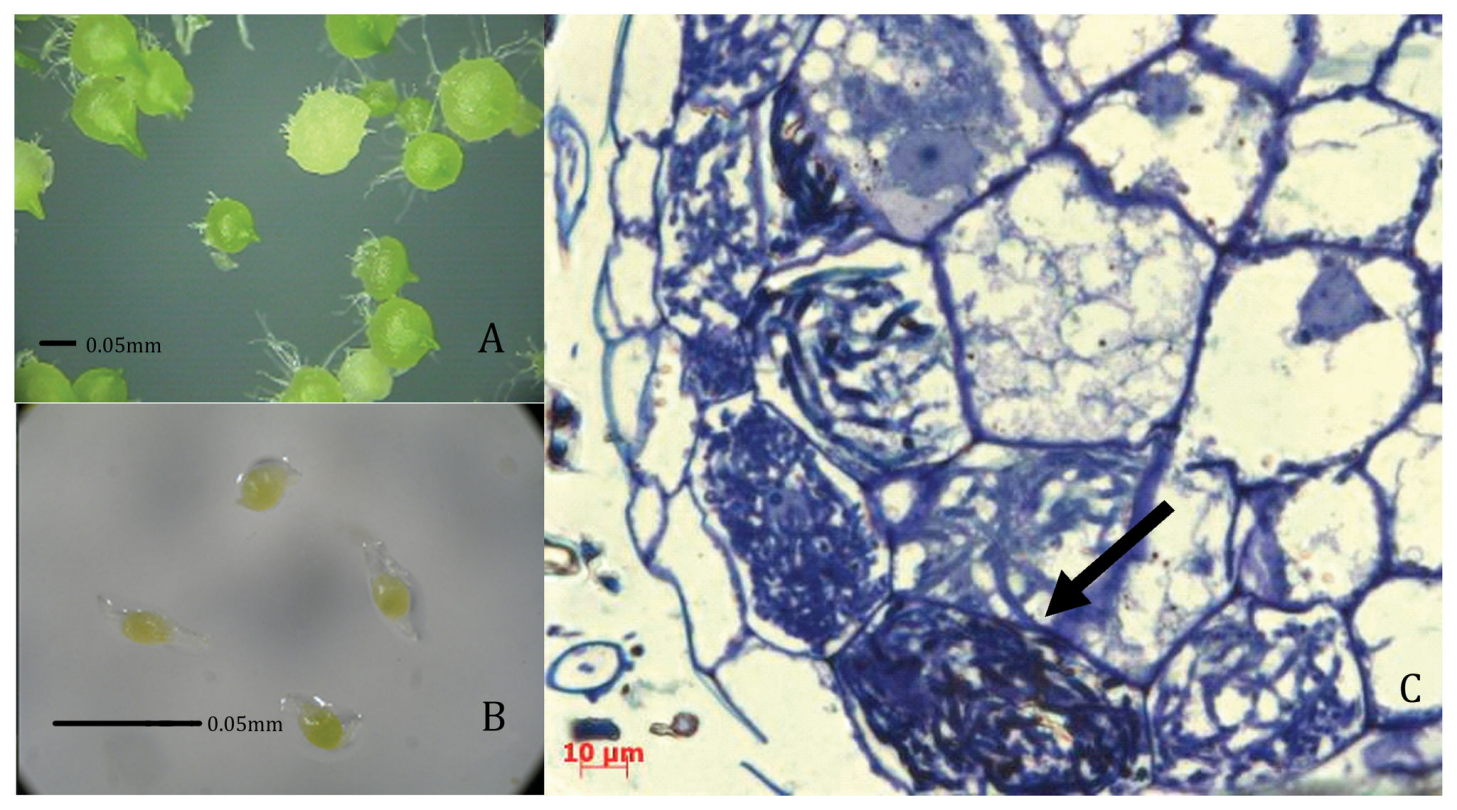
Observation of *D* *. officinale* Seeds cultured for 5 weeks (A and B) and invasion in SGS (C). Panels A and B show the pattern of third-stage SGS and UGS of *D* *. officinale* , respectively, using dissecting microscope. Scale bar (bold line), 0.05mm. Panel C: a semi-thin section was subjected to morphological examination using a Zeiss Axio Imager A1 microscope. The arrow indicates the pelotons of *Sebacina* sp. in *D* *. officinale* cell. Scale bar, 10 µm.

After cultivation for 5 weeks, seeds were examined using a dissecting microscope and were collected for further morphological assessment and RNA extraction. As shown in Figure 1A, almost all seeds cultivated with fungi germinated to the third stage. In contrast, all seeds cultivated without fungi remained un-germinated (Figure 1B). The germinated seeds infected with fungi were examined using semi-thin sections [28]. In the third-stage-germinated seeds, hyphae and mycelia knots convolved to form pelotons in plant cells, thereby indicating that a symbiotic relationship had been established (Figure 1C).

### Sequence assembly and EST annotation

In total, 1565 positive clones were sequenced, and ESTs containing no insert sequence or inserts shorter than 100 bp were removed prior to cluster analysis. A total of 1437 ESTs were obtained, with an average length of 518 bp. The remaining ESTs were aligned and assembled into 172 contigs (average length of 569 bp; range: 179-2202 bp) and 902 singletons (average length of 509 bp; range: 110-1096 bp). The ESTs clusters were divided into seven classes, including singles with one EST. Accordingly, 121 contigs containing 2 ESTs comprised the greatest proportion of the total accepted ESTs with the expression of singletons. We found 50 contigs containing ESTs ranging from 3 to 20 and one contig containing 68 ESTs, thereby suggesting a high level of transcription associated with the corresponding genes.

Genes for which the expression was altered at stage 3 symbiotic germination were identified. A total of 1074 Unigenes were subjected to BLASTX analysis for a homologous search against the NR database in GenBank. Of these, 777 Unigenes (70.08%) displayed at least one significant alignment with an existing gene in the database (E-value cutoff, e^-5^), and the remaining 297 Unigenes (29.92%) did not match any known sequence. These matched ESTs were named according to homologous sequences in the NCBI database and divided into two sets: *Dendrobium* and *Sebacina*. According to the results, 579 differentially expressed genes were identified in *Dendrobium* , and of these, 416 Unigenes had a known function. Based on comparison with genes expressed in UGS, a portion of genes were induced by OM symbiosis, including those in response to OM fungal signals and plant pathogens. The other genes were related to seed germination, including many that have been associated with carbon and nitrogen metabolism. A total of 198 Unigenes were identified in *Sebacina*; of these, 155 matched a known function. Most of these genes were involved in membrane transport and mineral nutrition metabolism.

### Functional classification of *D* *. officinale* subtractive ESTs

**Figure 2:**
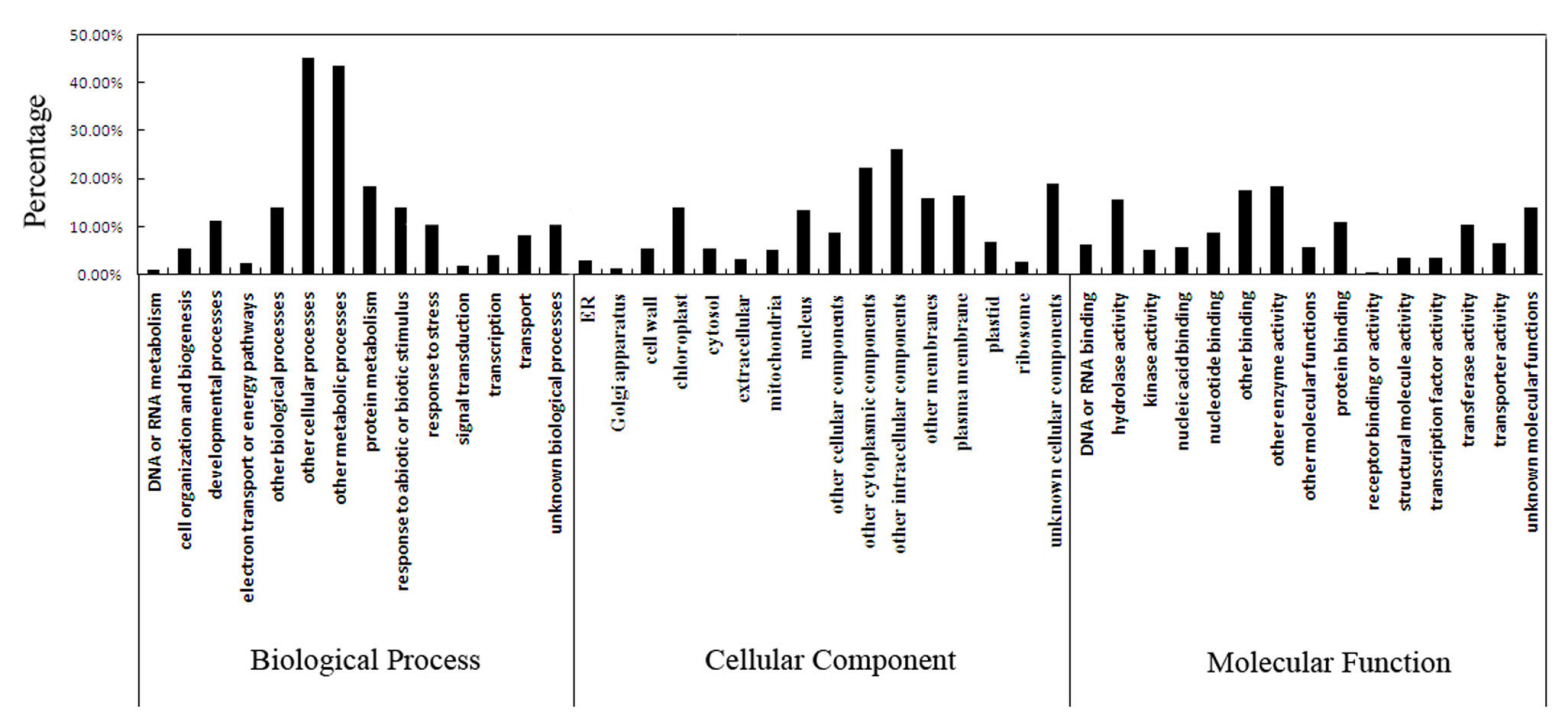
Representation of gene ontology assignments for plant homologous ESTs derived from SSH library. The GO slim Classification for Plants Developed at TAIR was used to functionally characterize the ESTs.

GO annotation was used to provide descriptions of gene products associated with molecular functions, cellular components and biological processes [29]. To determine the possible functions, we used the GO classification system for plants developed at TAIR, which is based on the *Arabidopsis thaliana* genome (http:// www.arabidopsis.org/help/helppages/go_slim_help.jsp). The annotations of unique sequences for *D* *. officinale* , for which GO categories could be assigned, were most frequently associated with biological processes followed by cellular components and molecular functions. Figure 2 shows the percentages of transcript models of subtractive ESTs for *D* *. officinale* in the 3 GO categories. These ESTs were classified into 14 categories of biological processes, primarily protein metabolism (18.42%), response to abiotic or biotic stimuli (14.11%), response to stress (13.43%) and unclassified processes. Plasma membrane (16.18%) and chloroplast (13.6%) were the most frequently represented categories in cellular components other than unclassified components. The largest proportion of molecular function ESTs were categorized as hydrolase activity (15.49%), protein binding (10.67%) and unclassified molecular functions.

**Figure 3:**
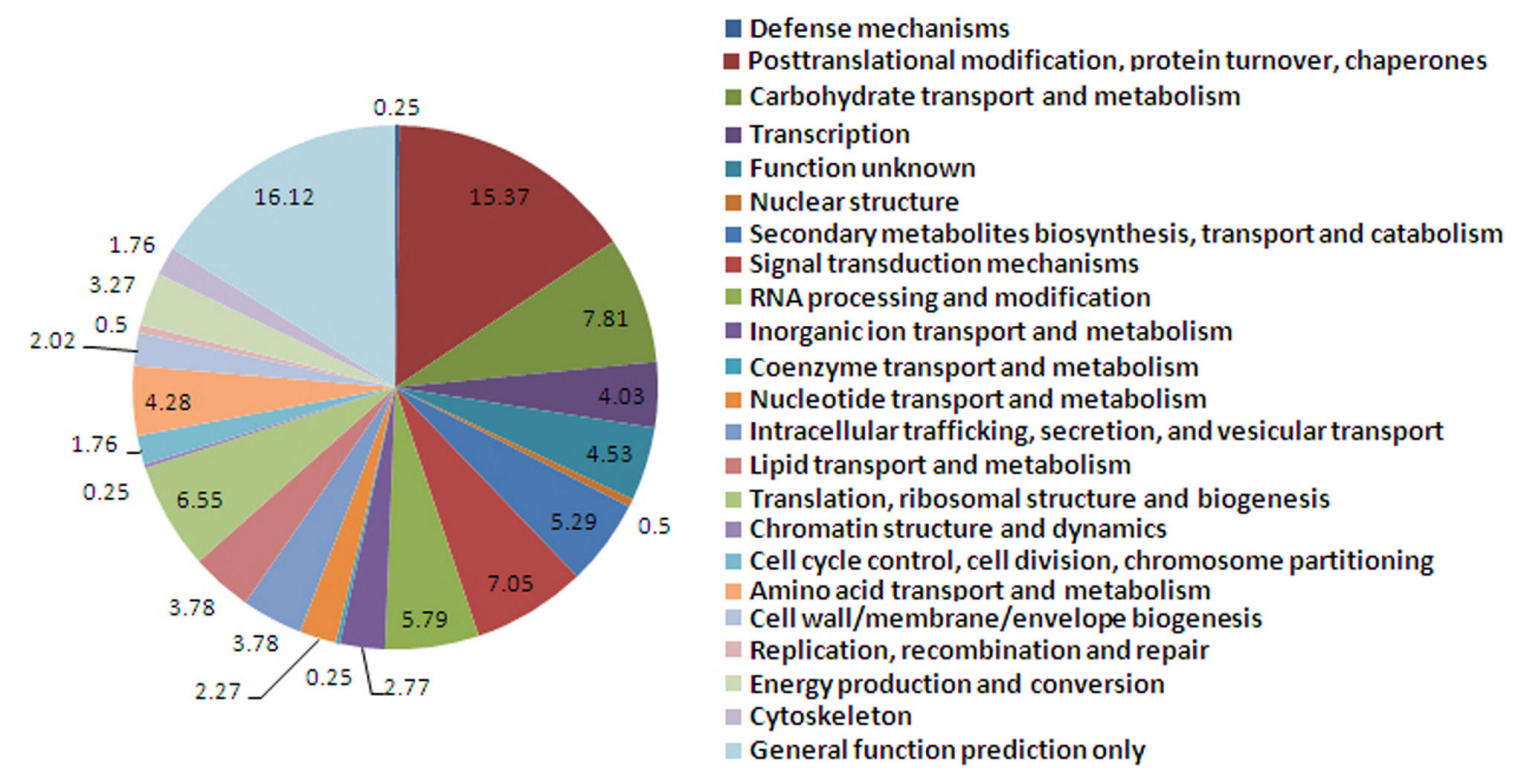
The functional classification of proteins deduced from plant homologous Unigenes in the SSH library using COGs.

Clusters of Orthologous Groups of proteins (COGs) were delineated by comparing protein sequences encoded in complete genomes, representing major phylogenetic lineages. COGs can predict individual protein features and the whole-genome protein function. The tagged proteins can be clustered into COGs comparing with all proteins in the COGs. To classify the possible functions of deduced proteins annotated by the SWISS-PROT database, we searched the COGs database for plant homologous proteins. Among all deduced plant homologous proteins, 369 displayed significant hit with COGs database, including 23 functional groups (Figure 3). These results show that the largest proportion of functions were protein metabolisms (15.37%), carbohydrate transport and metabolism (7.81%) and signal transduction (7.05%). Such categories as secondary metabolite biosynthesis, transport and catabolism (5.29%) and lipid transport and metabolism (3.78%) also represented a higher proportion than other classifications, thereby reflecting the high level of activity possibly linked to seed germination.

The unique sequences were assigned to KEGG annotations, which provide an alternative functional annotation of genes according to their associated biochemical pathways [25] based on sequence similarity searches against the KEGG database (Http://www.genome.jp/kegg/pathway.html). Unigenes of *D* *. offcinale* mapped to the KEGG pathways are shown in Table 1. Among these, 100 Unigenes were related to global maps, 170 corresponded to metabolism, 41 mapped to genetic information processing, 10 belonged to cellular processes and 8 were classified as organismal systems. Unigenes mapped to metabolism were the most abundant, which were associated with carbohydrate metabolism, amino acid metabolism, lipid metabolism, energy metabolism and nucleotide metabolism, all of which were related to germination. Eight genes were involved in environmental adaption, and three of these, mitogen-activated protein kinase (MPK), *CDPK* and transcription factor *WRKY*, participated in plant–pathogen interaction pathways and are assumed to be relavant for orchid symbiotic interactions [30,31].

**Table 1:** Unigenes mapped in KEGG pathways.

KEGG Pathways	Sub-pathways of KEGG Pathways	Number of Unigenes
Global Map		100
	Metabolic Pathways	68
	Biosynthesis of Secondary Metabolites	32
Metabolism		170
	Carbohydrate Metabolism	62
	Energy Metabolism	18
	Lipid Metabolism	21
	Nucleotide Metabolism	11
	Amino Acid Metabolism	26
	Metabolism of Other Amino Acids	10
	Glycan Biosynthesis and Metabolism	10
	Metabolism of Cofactors and Vitamins	2
	Metabolism of Terpenoids and Polyketides	2
	Biosynthesis of Other Secondary Metabolites	8
Genetic Information Processing		41
	Transcription	7
	Translation	9
	Folding, Sorting and Degradation	24
	Replication and Repair	1
Cellular Processes		10
	Transport and Catabolism	10
Organismal Systems		8
	Environmental Adaptation	8

### Real-time qPCR analysis of symbiotic germination-related genes

Real-time qPCR was employed to experimentally verify the expression patterns of 15 differentially expressed genes based on their putative functions and the results of the SSH library. Expression analysis was used to verify the validity of stage 3 symbiotic germination-related ESTs and to more precisely determine the timing of expression of the filtered genes of interest. As shown in Figure S1, all selected genes, including those encoding UDP-glucosyltransferase (17.5-fold), β-glucosidase (57.20-fold), immediate-early fungal elicitor (9.28-fold), chitinase (33.94-fold), leucine-rich repeat receptor kinase (LRR-RK) (29.37-fold), β-1, 3-glucanase (23.67-fold), cysteine protease (23.67-fold), NAC transcription factor (83.34-fold), CDPK32-like (26.69-fold), agglutinin (4.43-fold), auxin-responsive protein (3.75-fold), catalase (3.24-fold), early nodulin putative (5.39-fold), cation exchanger (8.49-fold) and CDPK1 (6.76-fold), were verified to be upregulated in SGS.

### Characterization and sequence analysis of full-length DoCDPK1 and DoCDPK32-like

CDPKs are the largest group of complex calcium sensors, and they play a key role in a variety of biological processes, including plant growth and development, abiotic and biotic stress adaptations, and seed germination, via calcium signaling pathways [32]. To gain further insight into the role of CDPKs in *D* *. officinale* symbiotic gemination, the corresponding full-length cDNAs of two CDPKs were isolated via RACE assay. *DoCDPK1* ( *D* *. officinale* *CDPK1*) and *DoCDPK32-like* ( *D* *. officinale* *CDPK32-like*), according to the nomenclature of reference [33], were obtained via 3'RACE and 5'RACE, and positive clones were sequenced. Trimmed out of vectors using VecScreen followed by re-assembling, two full-length cDNA (2236 bp and 2124 bp) were obtained. To confirm the full-length cDNA sequences, PCR was performed with primers (DoCDPK1-orfF/R; DoCDPK32-like-orfF/R) (Table S1) spanning ORFs. Single bands of 1.9 kb and 1.85 kb for *DoCDPK1* and *DoCDPK32-like*, respectively, were observed via agarose gel electrophoresis. After cloning and sequencing, the products were verified to be consistent with the spliced sequences described above. The two sequences were deposited into GenBank under the accession numbers JX002662 (*DoCDPK1*) and JQ927225 (*DoCDPK32-like*), respectively. *DoCDPK1* contained a 1578 bp ORF with a 5'-UTR (un-translated region) of 266 bp and a 3'-UTR of 392 bp. The ORF of *DoCDPK32-like* was 1407 bp in size and comprised of a 5'-UTR of 442 bp and a 3'-UTR of 275 bp. Each sequence contained a eukaryotes polyA tail and the typical Kozak consensus sequence (A/GNNATGG) located at the beginning of the coding region. Using Compute PI/MW, the predicted molecular weight of DoCDPK1 and DoCDPK32-like were 58.34 KDa and 51.48 KDa, respectively, and the prediction of *pI* values was 5.94 and 6.11, respectively. Both deduced proteins contain a conserved serine/threonine protein kinase catalytic domain. DoCDPK1 contains the typical four EF-hand motifs. In contrast, this motif was not found in DoCDPK32-like.

### Multiple Sequence Alignment, Phylogenetic relationships and tissue expression pattern analysis of DoCDPK1 and DoCDPK32-like

DoCDPK1 and DoCDPK32-like were aligned to several other plant CDPKs using MegAlign in DNAStar 6.0 (Figure S2). DoCDPK1 had the highest level of homology (86%), with PgCDPK1 (ACY78680) of *Panax Ginseng* followed by *Zea mays* ZmCDPK2（NP_001105542）and *Hevea* *Brasiliensis* HbCDPK1 (ACB71246) with 83% identity. DoCDPK32-like displayed the highest level of homology with *Vitis vinifera* VvCDPK25-like (47%), while the homology was 42% with the other three proteins. BLAST searches and subsequent domain analysis also revealed that each of these CDPKs displayed similar domain compositions. The protein kinase ATP-binding signature and S-TKc active-site signature of all sequences were well conserved. Regarding the protein kinase ATP-binding signature, each sequence started with Leu–Gly and ended with Lys. Similarly with respect to the S-TKc active-site signature, the active-site was Asp for all sequences.

For phylogenetic analysis, DoCDPK1, DoCDPK32-like and sixty-four homologous CDPKs were aligned using ClustalW in Mega 4.0 (Figure S3). The results indicate that DoCDPK1 belongs to group II-a (13), it also clustered with *Oryza sativa* OsCDPK19 in the same branch and was phylogenetically closest to the AtCDPK9 and AtCDPK33 branches. DoCDPK32-like grouped with GmCDPK32-like and was distant from other plant CDPKs groups.

Real-time qPCR was used to profile *DoCDPK1 and DoCDPK32-like* (Figure S4). The results show that both genes are constitutively expressed. The highest level of the *DoCDPK1* transcript level was found in leaf samples, with 8.54-fold expression, whereas *DoCDPK32-like* was expressed 17.34-fold higher in root samples.

## Discussion

Although widespread investigations of orchid seedling germination have been performed for well over a century, the mechanism of this germination at the molecular level remains unclear. Much less is known regarding the genetically controlled checkpoints that facilitate or restrict symbiotic development and the plant and fungal genes that are involved in the process. This work reflects a preliminary characterization and provides the first investigation of orchid symbiotic germination related genes, especially focusing on plant response genes. Compared with *Sebacina*, the sequence in *Dendrobium* is well known and the plant genotype can affect the extent of the fungi colonization and the response. A variety of plant homologous ESTs were classified and most were assigned as germination-related and symbiosis-related. These Unigenes were identified, annotated and found to participate in many biological processes, such as transcription, transduction, transport and metabolism. By analyzing the annotation of Unigenes mapped onto KEGG database metabolism pathways, the most abundant subset belonged to carbohydrate metabolism followed by amino acid and lipid metabolism (Table 1). Two carbohydrate metabolism Unigenes encoding UDP-glucosyltransferase and β-glucosidase were significantly upregulated by 17.35- and 57.20-fold in SGS, respectively. In the current report, various genes involved in transport processes, including carbohydrate transporter, cation transporters, ATP/ADP-transporters and nucleoside transporters, were determined to be upregulated, thereby indicating that a soluble or insoluble source of carbohydrate, nitrogen and phosphorus translocated from the fungus to the plant. This observation suggests that a symbiotic relationship was established and nutrients accumulated in the plant for the next germination stage.

In this work, we paid close attention to genes putatively involved in signaling, and several genes associated with signal transduction were identified during the course of OM symbiosis. The transcript encoding LRR-RK, which has been reported to be involved in plant defense response and shoot development [34], displayed a 29.37-fold increase in SGS. CDPKs and MPKs are two common protein kinases involved in diverse signal regulation pathways. MPKs, which mediate intracellular transmission and amplification of extracellular stimuli, have been demonstrated to be essential for plant-microbe symbiosis [35]. CDPKs have also been reported to be involved in symbiotic systems and seed germination. *MtCDPK3* was confirmed to be upregulated during nodulation in *M. truncatula* [36]. *OsCDPK18* was shown to be transcriptionally activated in response to inoculation with *Glomus intraradices* [37]. ABA-stimulated CDPK, *ACDPK1*, derived from grapes, promotes plant growth and enhances ABA sensitivity in seed germination [38]. Moreover, *AtCDPK32* has been shown to be involved in ABA-regulated seed germination [39]. Real-time qPCR analysises indicate that the two CDPKs cloned in the current study display a tissue-specific expression pattern and are both upregulated in SGS. This result suggests that these genes may be induced by fungi and may play a vital role in the regulation of *D* *. officinal* symbiosis.

Symbiosis in which fungus infects and colonizes the plant would trigger a defense response. The production of hydrolytic enzymes by the plant may be important for lysis of the fungal cell wall. Chitinase and β-glucanase are two common enzymes often implicated in the response of plants to parasite infection [6], and their expression can be induced by fungi [40,41]. Fungal elicitors are proteins that induce necrosis in plants, thereby causing the plant to become resistant to pathogens [42]. Genes encoding immediate-early fungal elicitor proteins, chitinase and beta-1, 3-glucanase, were selected and their expression levels were elevated 2-fold in SGS compared with UGS. This situation is similar to AM symbiotic relationships [6]. A phytohormone-related gene encoding auxin response protein was also discovered and seems to be upregulated in SGS. The auxin response protein had been reported to function in defense response in *Pinus* *pinaster* roots against fungal pathogen attacks [43].

Two pathways in which genes may participate in the regulation of the plant–fungus interaction are shown in Figure S5. The polyamines spermidine (Spd), spermine (Spm), and their precursor, putrescine (Put), are low-molecular-weight aliphatic amines produced ubiquitously in many physiological processes in both eukaryotes and prokaryotes, especially higher plants [44]. Kytöviita et al. [45] have reported that polyamines are involved in establishing and maintaining mycorrhizal symbiosis. The concentration of free polyamines in roots is altered by inoculating with ectomycorrhizal (ECM) fungi, and the supply of specific polyamines enhances the formation of both the ECM [46] and AM [47]. In our work, three major enzymes involved in polyamine synthesis pathways were identified among the differentially expressed genes, including arginine decarboxylase (ADC), S-adenosylmethionine decarboxylase (SAMDC) and spermidine synthase (SPDS) (Figure S5A). These genes may collectively function in polyamine circulation during OM symbiosis. Another pathway associated with plant–pathogen interactions is activated by Ca^2+^ according to KEGG ath04626, including *CDPK, MPK* and *WRKY* for instance (Figure S5B).

Compared with *Dendrobium* derived ESTs, relatively few genes were identified from *Sebacina*. A majority of these ESTs are involved in membrane transport and mineral nutrition metabolism, including H^+^-ATPases, sucrose transporter, protein transporter, inorganic phosphate transporter and lipase. H^+^-ATPases are reported to play a key role in establishing the electrochemical gradient required for the transfer of nutrients across the plasma membrane in AM symbiosis [48]. As the expression of these genes is altered during the interaction, they should be essential for fungus to establish a compatible relationship with the plant. The understanding of gene expression regulation in OM fungi is much more limited; therefore, more studies are required to test this hypothesis and determine these genes’ functions in OM symbiosis.

In this study, we successfully used a combination of SSH library and real-time qPCR techniques to determine gene expression patterns during orchid symbiotic germination. These genes and their putative functions provide insight into *Dendrobium* symbiotic seed germination and provide candidate genes for further investigations of the molecular events that control OM symbiosis.

## Supporting Information

Figure S1 **Expression patterns of fifteen subtractive plant homologous genes from SSH library using qPCR analyses.** Dark grey columns represent the RQ in SGS; light grey columns indicate the RQ in UGS. All standards were run in duplicate and samples were run in triplicate. 1: NAC transcription factor; 2: cation exchanger; 3: auxin-responsive protein; 4: LRR; 5: cysteine protease; 6: chitinase; 7: β-1,3-glucanase; 8: catalase; 9: UDPG; 10: CDPK1; 11: immediate-early fungal elicitor; 12: agglutinin; 13: early nodulin putative; 14: β-glucosidase; 15: CDPK32-like
(TIF)

Figure S2 **Multiple sequence alignments of DoCDPK1 (A), DoCDPK32-like (B) and CDPKs proteins from other plants.** Thick lines indicate the S-TKc domain ; Dot lines indicate the four Ca^2+^ binding EF-hand motifs. At: *Arabidopsis thaliana*; Do: *Dendrobium* *officinale* ; Gm: *Glycine max*; Hb: *Hevea* *brasiliensis* ; Os: *Oryza sativa*; Pg: *Peanaxginseng* ; St: *Solanum tuberosum*; Vv: *Vitis vinifera*; Zm: *Zea mays*.
(TIF)

Figure S3 **Phylogenetic tree of DoCDPK1 and DoCDPK32-like with CDPKs genes from other plants.** At: *Arabidopsis thaliana*; Do: *Dendrobium* *officinale* ; Gm: *Glycine max*; Os: *Oryza sativa*
(TIF)

Figure S4 **Tissue-specific expression patterns of *DoCDPK1* and *DoCDPK32-like* genes using real-time qPCR analyses (A and B, respectively).**
(TIF)

Figure S5 **Genes involved in polyamine synthesis pathways (A) and plant–pathogen interaction pathways (B).** Shadow genes represent those obtained from the SSH library. SAM: S-adenosylmethionine; dcSAM: decarboxylated S-adenosylmethionine; SAMDC: S-adenosylmethionine decarboxylase; ADC: argininedecarboxylase; SPDS: spermidine synthase; SPMS: spermine synthase; CDPK: calcium-dependent protein kinase; Rboh: respiratory burst oxidase homo-logs; ROS: reactive oxygen species; MPK: mitogen-activated protein kinases
(TIF)

Table S1 **Gene specific primers for real-time qPCR.**
(DOC)
