# Supplementary figures and images for: ESTs Analysis Reveals Putative Genes Involved in Symbiotic Seed Germination in Dendrobium officinale
Source: PLoS One. 2013 Aug 13;8(8):e72705. doi: 10.1371/journal.pone.0072705 (PMC3742586; doi:10.1371/journal.pone.0072705)

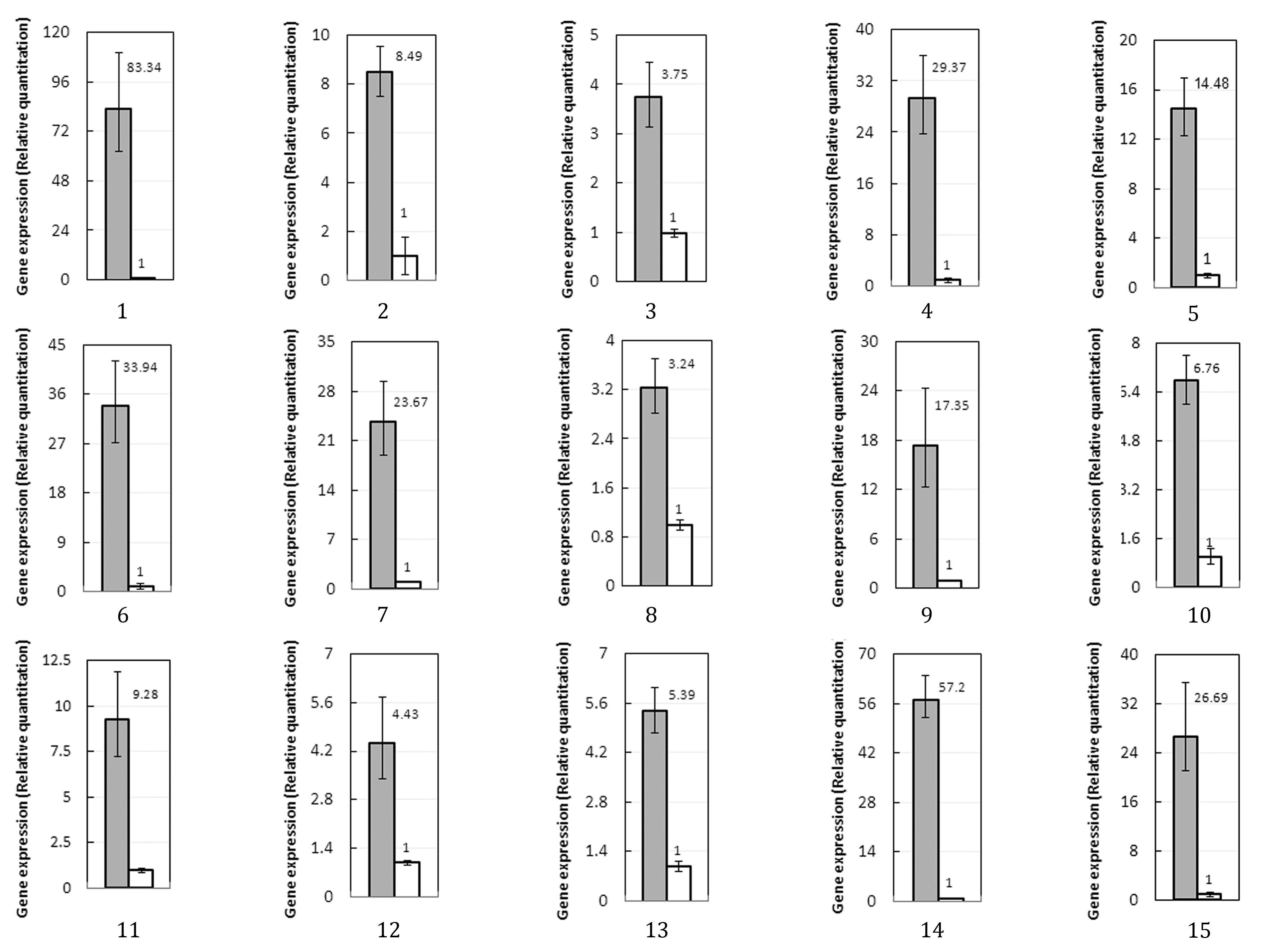

Supplement: Figure S1 — Expression patterns of fifteen subtractive plant homologous genes from SSH library using qPCR analyses. Dark grey columns represent the RQ in SGS; light grey columns indicate the RQ in UGS. All standards were run in duplicate and samples were run in triplicate. 1: NAC transcription factor; 2: cation exchanger; 3: auxin-responsive protein; 4: LRR; 5: cysteine protease; 6: chitinase; 7: β-1,3-glucanase; 8: catalase; 9: UDPG; 10: CDPK1; 11: immediate-early fungal elicitor; 12: agglutinin; 13: early nodulin putative; 14: β-glucosidase; 15: CDPK32-like (TIF) [file pone.0072705.s001.tif]

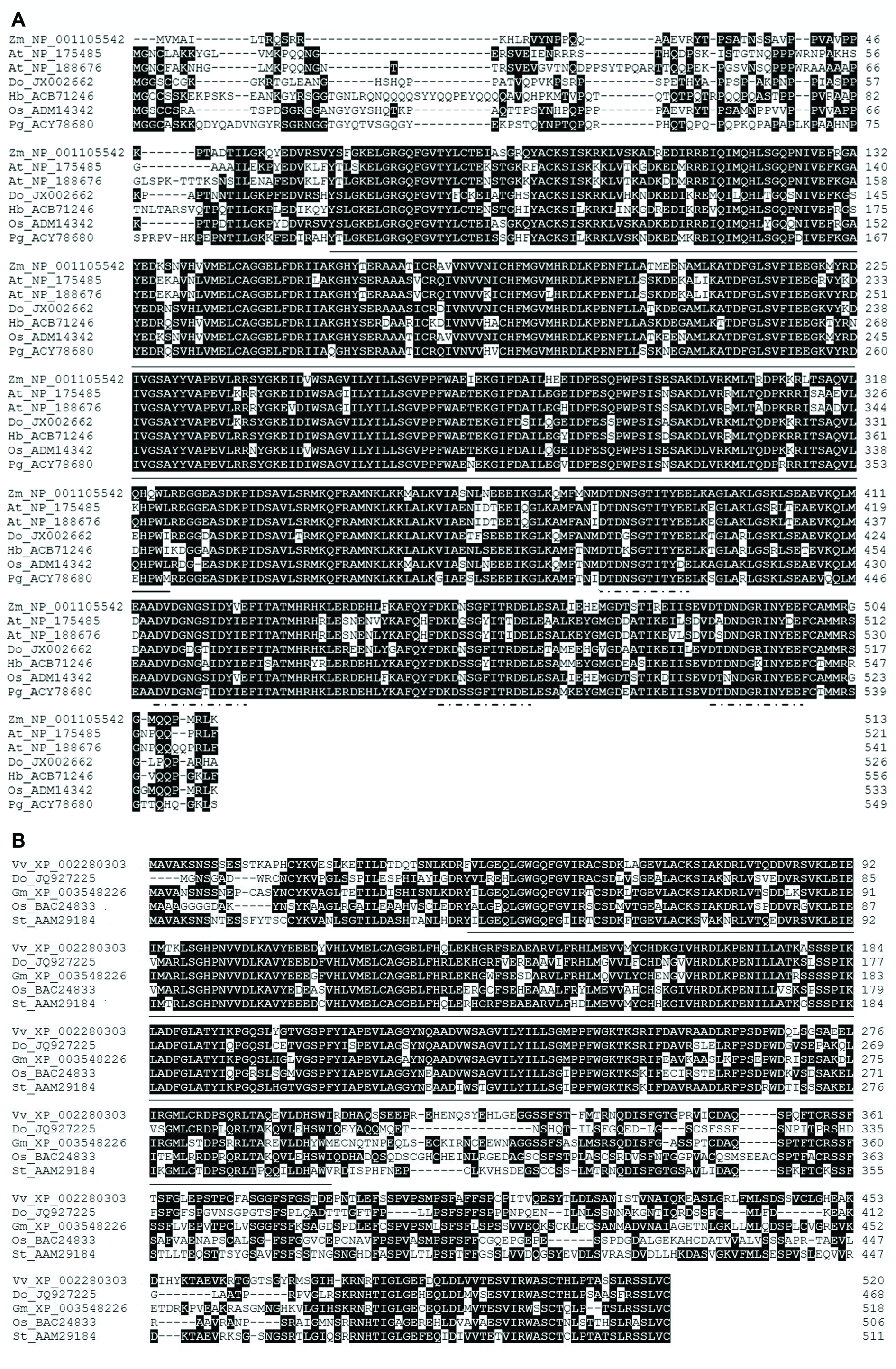

Supplement: Figure S2 — Multiple sequence alignments of DoCDPK1 (A), DoCDPK32-like (B) and CDPKs proteins from other plants. Thick lines indicate the S-TKc domain ; Dot lines indicate the four Ca2+ binding EF-hand motifs. At: Arabidopsis thaliana; Do: Dendrobium officinale ; Gm: Glycine max; Hb: Hevea brasiliensis ; Os: Oryza sativa; Pg: Peanaxginseng ; St: Solanum tuberosum; Vv: Vitis vinifera; Zm: Zea mays. (TIF) [file pone.0072705.s002.tif]

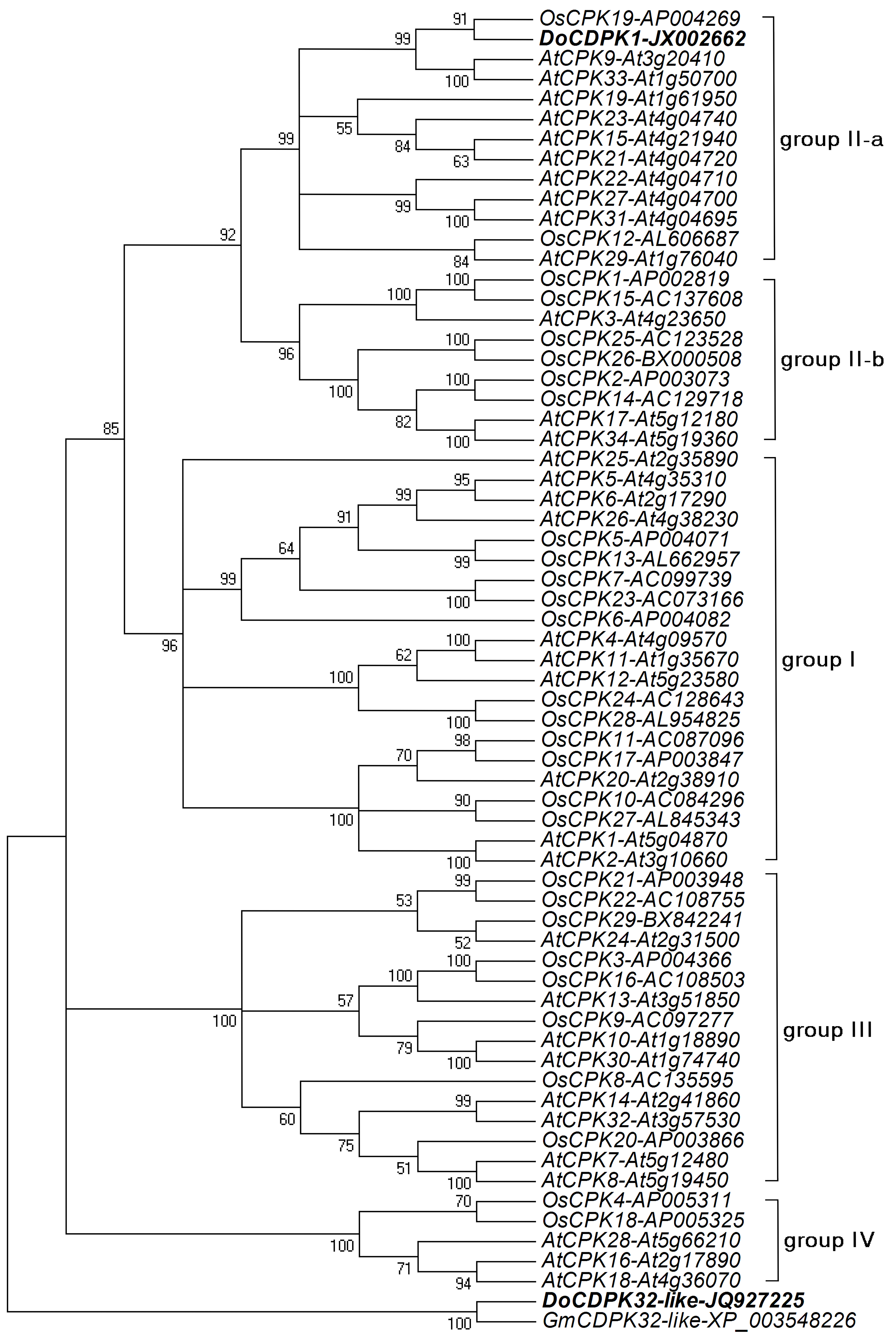

Supplement: Figure S3 — Phylogenetic tree of DoCDPK1 and DoCDPK32-like with CDPKs genes from other plants. At: Arabidopsis thaliana; Do: Dendrobium officinale ; Gm: Glycine max; Os: Oryza sativa (TIF) [file pone.0072705.s003.tif]

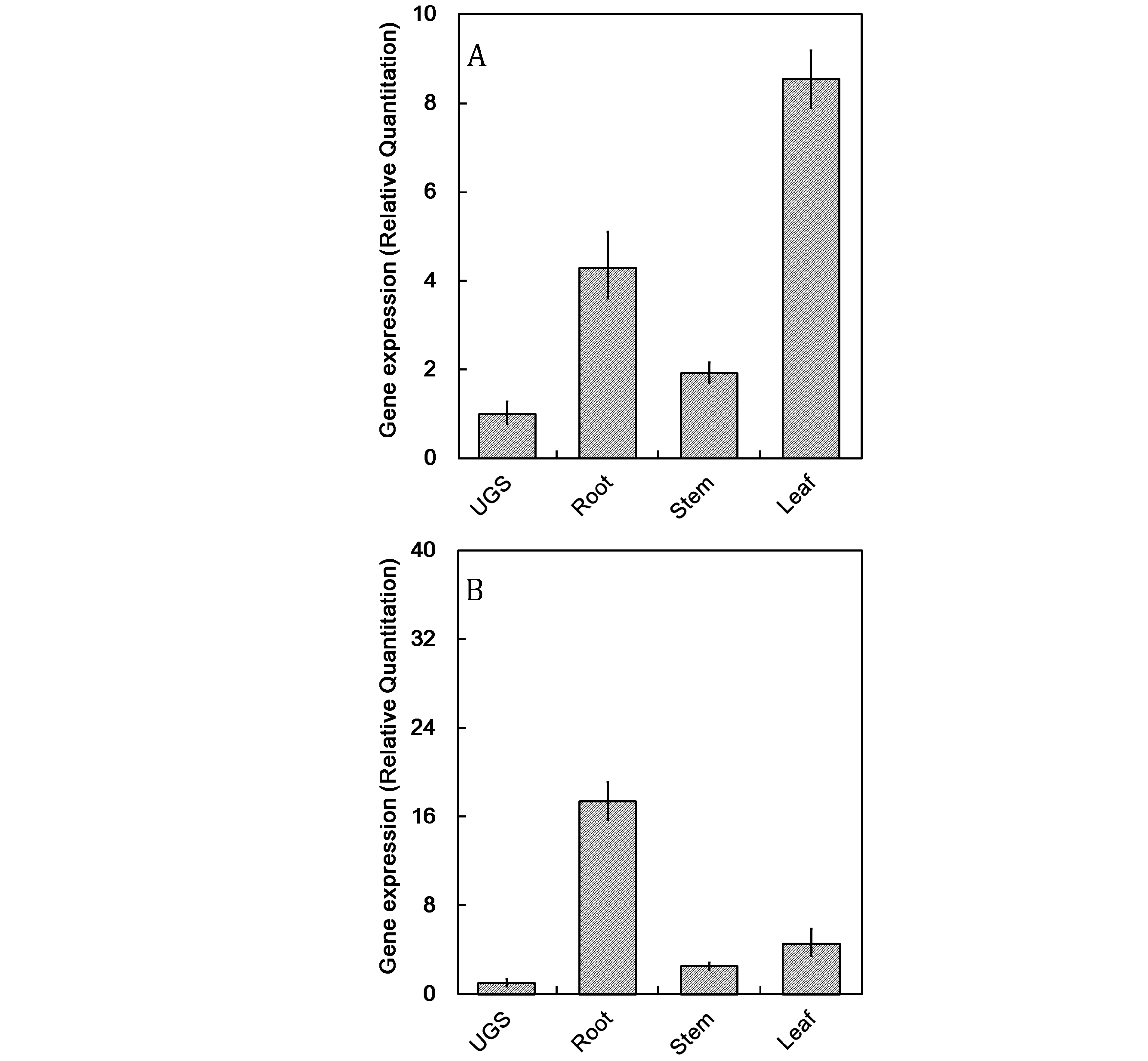

Supplement: Figure S4 — Tissue-specific expression patterns of DoCDPK1 and DoCDPK32-like genes using real-time qPCR analyses (A and B, respectively). (TIF) [file pone.0072705.s004.tif]

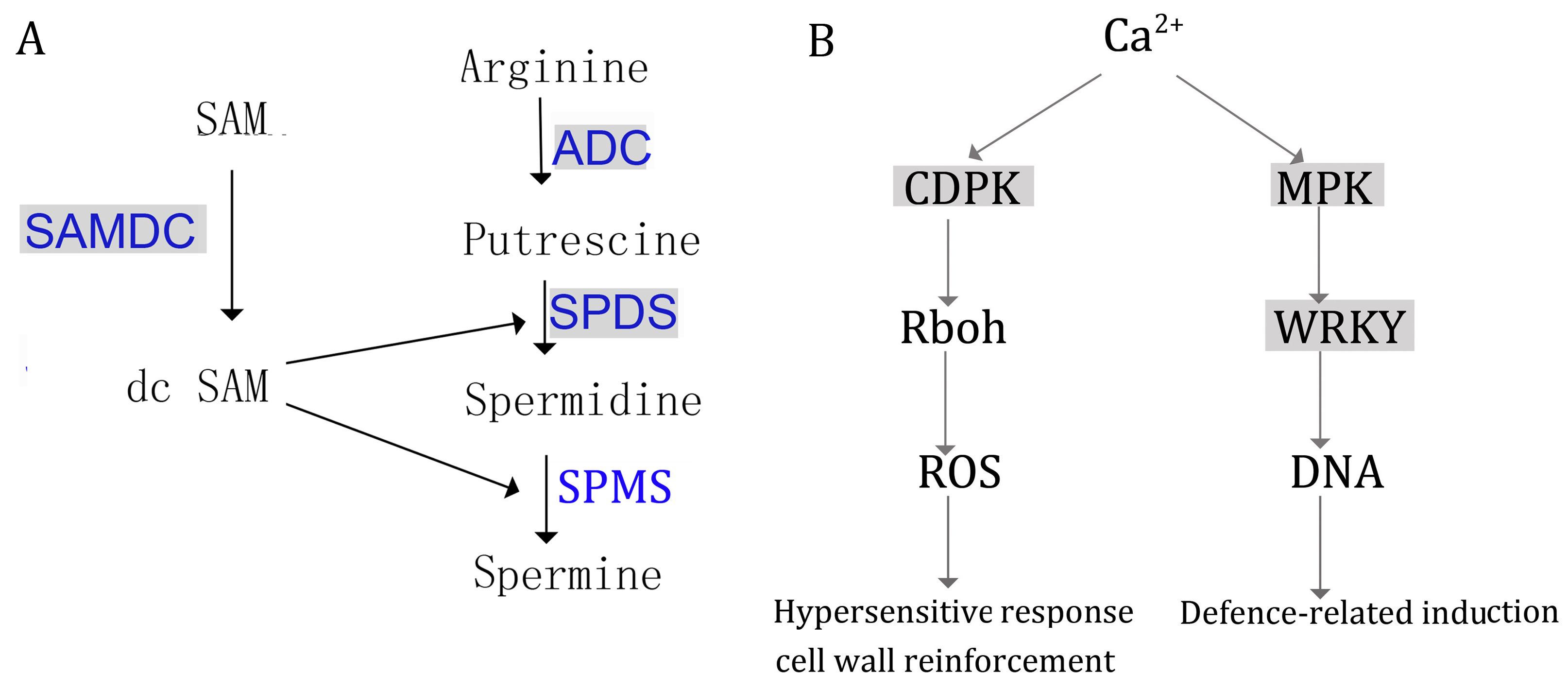

Supplement: Figure S5 — Genes involved in polyamine synthesis pathways (A) and plant–pathogen interaction pathways (B). Shadow genes represent those obtained from the SSH library. SAM: S-adenosylmethionine; dcSAM: decarboxylated S-adenosylmethionine; SAMDC: S-adenosylmethionine decarboxylase; ADC: argininedecarboxylase; SPDS: spermidine synthase; SPMS: spermine synthase; CDPK: calcium-dependent protein kinase; Rboh: respiratory burst oxidase homo-logs; ROS: reactive oxygen species; MPK: mitogen-activated protein kinases (TIF) [file pone.0072705.s005.tif]
