# Supplementary material for: ESTs Analysis Reveals Putative Genes Involved in Symbiotic Seed Germination in Dendrobium officinale
Source: PLoS One. 2013 Aug 13;8(8):e72705. doi: 10.1371/journal.pone.0072705 (PMC3742586; doi:10.1371/journal.pone.0072705)
Supplement: Table S1 — Gene specific primers for real-time qPCR. (DOC) [file pone.0072705.s006.doc]

**Table S1 Gene specific primers for real-time qPCR**

| EST No. | Primer sequence（5'-3'） | Tm（℃） | Amplicon size（bp） |
| --- | --- | --- | --- |
| Do-1986-rtF | TTGGAGTTTGCGTGGGGT | 59.1 | 107 |
| Do-1986-rtR | TCAATGAAATCTGGCGGC | 57.3 |  |
| Do-697-rtF | ATGAAGGGCATTGAAGAAGCA | 59.9 | 129 |
| Do-697-rtR | AGAGGTGGCTGTCCCAAAGAG | 60.7 |  |
| Do-1670-rtF | CGATCACCGGAACCCTAA | 56.2 | 418 |
| Do-1670-rtR | TCTGCCTCTGAACTCTCACATT | 56.5 |  |
| Do-131-rtF | TGCGACAAGGTGGTATAGGGC | 62.4 | 114 |
| Do-131-rtR | TTCCCAGTCAGAACCTCAGCAA | 62.1 |  |
| Do-1357-rtF | TTCCCAATACCGCCAACT | 55.9 | 395 |
| Do-1357-rtR | CAAATAGCAGCAAACAAGATGA | 56.2 |  |
| Do-992-rtF | TTGTCATGGCGGTTAGGC | 55.6 | 104 |
| Do-992-rtR | TCCAATGGCGGAGGAAGG | 61.1 |  |
| Do-1417-rtF | AGTGGCTTCGAGGTTTGCA | 58.9 | 462 |
| Do-1417-rtR | TCGCCTTCTCCTTCGTGTT | 58.0 |  |
| Do-C18-rtF | GGGGATAAGCGAATCAAAGA | 57.0 | 208 |
| Do-C18-rtR | TGCCGAAAGAGCAGAGGAA | 59.1 |  |
| Do-1422-rtF | ATCAATGACGACTGCGGC | 57.0 | 255 |
| Do-1422-rtR | CGAGAAGACATCTGGTGCG | 56.9 |  |
| Do-520-rtF | CTTCTCGCTCCTCTCCTCCG | 61.4 | 185 |
| Do-520-rtR | GGTTTAGACCTACCACTTCGCC | 59.9 |  |
| Do-1930-rtF | GGAAGATAAATAAATGCGGACA | 56.9 | 256 |
| Do-1930-rtR | CCCCAAAAACAGCAAGACC | 57.8 |  |
| Do-1561-rtF | CTCGGCTACTCGAACCTCTT | 56.4 | 382 |
| Do-1561-rtR | CCATCAATCTCCAATCCATCTC | 58.4 |  |
| *GAPDH*-rtF | CAAGGACTGGAGAGGTGGAAGA | 60.7 | 102 |
| *GAPDH*-rtR | GACCTGCTGTCACCCAAGAAGT | 60.6 |  |
| DoCDPK1-F1 | GAAGGCGGCGACGCATCTGATAAACC | 73.8 |  |
| DoCDPK1-R1 | GTTTATCAGATGCGTCGCCGCCTTC | 71.0 |  |
| DoCDPK32-like-F1 | TCGGCCTCGCTACTTACATCCAAC | 66.7 |  |
| DoCDPK32-like -R1 | TCTGCCCAGGTTGGATGTAAGTAGCG | 69.6 |  |
| DoCDPK1-orfF | CCTGTTCCGTTTCATTGTTT | 55.1 | 1899 |
| DoCDPK1-orfR | TCACTTTTTGCATTTTCTTCC | 55.1 |  |
| DoCDPK32-like -orfF | GCTTCCATCTCCCGTGTC | 55.2 | 1850 |
| DoCDPK32-like-orfR | CCCCAGCTCTTCATTCACC | 57.4 |  |
| DoCDPK1-rtF | GGCAGAAACCGAAAAGGG | 57.8 | 129 |
| DoCDPK1-rtR | TTCGGGTCCTGGGTAAGC | 57.9 |  |
| DoCDPK32-like -rtF | CAGGCGGCAGATGTTTGG | 60.3 | 166 |
| DoCDPK32-like -rtR | ATTGCTTTGCGGGTTCAG | 56.7 |  |
